# Evaluation of skin pigmentation effect on photoplethysmography signals using a vascular finger phantom with tunable optical and mechanical properties

**DOI:** 10.1117/1.JBO.30.11.117002

**Published:** 2025-11-25

**Authors:** Laura Osorio-Sanchez, James M. May, Panicos Kyriacou

**Affiliations:** City St. George’s, University of London, Research Centre for Biomedical Engineering, London, United Kingdom

**Keywords:** photoplethysmography, tissue phantoms, racial bias, skin pigmentation, optical characterization, light-tissue interaction

## Abstract

**Significance:**

Photoplethysmography (PPG) is a widely used optical technique for the noninvasive monitoring of cardiovascular parameters. However, its accuracy may be affected by variations in skin pigmentation due to the strong absorption properties of melanin, particularly at visible wavelengths.

**Aim:**

We aimed to investigate how skin tone influences PPG signal signals by developing a pulsatile vascular finger phantom with interchangeable skin layers, characterizing their optical properties across green, red, and infrared wavelengths and evaluating their impact on PPG signal features

**Approach:**

The finger phantom included three optically characterized, interchangeable skin layers representing pale, medium, and dark tones, as well as a custom-made silicone vessel embedded in an anatomically and mechanically characterized structure. PPG signals were recorded in reflectance mode using a custom-made finger clip probe in an *in vitro* cardiovascular system. Signal features, including signal-to-noise ratio, peak-to-peak amplitude, and area under the curve, were analyzed.

**Results:**

Analysis revealed statistically significant differences (p<0.001) between skin tones, with signal degradation increasing with skin pigmentation.

**Conclusions:**

These findings suggest there is a measurable impact of skin pigmentation on the PPG signal and highlight the need for further research to improve the equity of light-based sensing technologies across all populations. We provide an advancement for future work in developing *in vitro* models to assess optical sensing performance across diverse skin tones.

## 1 Introduction

The impact of skin pigmentation on light-tissue interaction sensing applications, such as photoplethysmography (PPG) and pulse oximetry, is not a new concern, but it has gained increased attention during and after the COVID-19 pandemic. The higher mortality rate among individuals with darker skin raised concerns about whether skin color contributes to inequalities in pulse oximetry and other optical measurement techniques.^1^^–^^5^ Although some research has explored this topic, gaps remain in fully characterizing these effects and developing solutions to mitigate potential disparities. A more comprehensive understanding is essential to ensure equitable and accurate health monitoring for all individuals, emphasizing the need for continued research in this field.^5^^,^^6^

Despite their growing importance, optical sensing methods are influenced by several biological factors, with skin pigmentation being a particularly critical variable. Variations in melanin concentration can affect the way light is absorbed and scattered within the skin, potentially introducing biases in PPG and other light-based measurements.^5^ Darkly pigmented skin has higher melanin content, causing a greater attenuation of the incident light compared with lighter skin.^7^^,^^8^ This reduces the depth of light penetration and limits the accuracy of measurements, which is evidenced in several studies that suggest that pulse oximetry may be less reliable in individuals with darker skin tones,^1^^,^^9^^–^^21^ which has also been supported by an independent review from the UK government of the health impact of potential bias in medical devices.^3^

Several factors can impact the PPG signal, including low perfusion, ambient light, motion artifact, applied pressure, and skin color.^6^^,^^22^ *In vitro* phantom models offer controlled environments, making them an ideal approach for identifying performance issues, as they allow for controlled manipulation of variables that are difficult to regulate in *in vivo* studies. In this study, they are particularly valuable for isolating the effect of skin pigmentation on the PPG signal. The literature on phantom materials and fabrication methods is extensive, reflecting significant advancements in recent years. Various phantom designs have been developed to mimic human physiology, particularly for testing optical sensing systems such as PPG and oximetry, with silicone being the most commonly used material.^23^^,^^24^ Some approaches have been used to mimic skin pigmentation.^25^^–^^29^

Afshari et al.^25^ developed a phantom model to simulate cerebral oximetry and assess the impact of skin pigmentation on commercial cerebral oximeters. It incorporated an epidermal layer simulating varying melanin concentrations, using water-soluble nigrosine, nail polish remover, and titanium dioxide particles to adjust the optical properties. Building on this approach, Bhusal et al.^26^ created a tissue-mimicking finger phantom, integrating an epidermis layer fabricated using Afshari et al.’s method for simulating different skin tones. Jenne and Zappe^27^ developed a three-layer tissue-mimicking phantom for PPG applications, replicating the optical and mechanical properties of the epidermis, dermis, and hypodermis. Optical characteristics were adjusted using India ink, synthetic melanin, and titanium dioxide particles. Expandable channels were integrated into the model. Vogt et al.^28^ manufactured a breast phantom with swappable skin-mimicking layers to evaluate the impact of epidermal content on photoacoustic imaging devices. Alcohol-soluble nigrosine was used as the dye to mimic melanin absorption. Swamy et al.^29^ developed a test bench to evaluate commercial pulse oximeters, incorporating melanin filters to simulate a range of skin tones. These filters, made with M8631 synthetic melanin, were designed to attenuate red and infrared (IR) light, with red wavelengths experiencing greater attenuation than IR. Notably, only Jenne and Zappe^27^ and Vogt et al.^28^ included analysis of the green wavelength in their optical characterization, whereas the other studies focused primarily on red and IR.

Although recent research has produced valuable insights and promising phantom-based approaches, current models appear to fall short in fully replicating the complexity of biological tissue. An ideal phantom for evaluating the effects of skin pigmentation on PPG signals would require a multi-layered structure that closely mimics the anatomical composition of human skin and subcutaneous tissue, avoiding oversimplified representations. It should feature precisely tuned optical properties, with interchangeable layers representing a range of skin tones to simulate diverse pigmentation and its influence on light absorption and scattering. Realistic mechanical properties are essential to replicate the compliance of human tissue under pressure. To generate pulsatile signals, including expandable blood vessels, is fundamental for PPG measurements. Ideally, the phantom should incorporate the anatomical shape of the tissue being simulated. Finally, integrating a circulating fluid system, rather than a closed-end setup, would better replicate continuous blood flow and dynamic pulsations, offering a closer representation of physiological conditions. Importantly, to reflect common wearable device designs and expand upon previous work, the phantom should characterize multiple wavelengths, including green light, which is frequently used in wearable PPG sensors, enabling a more comprehensive and innovative evaluation of optical performance across different tissue types.

The purpose of the current study is to provide new knowledge and facilitate understanding of how skin pigmentation affects the PPG signal and the implications this potentially has on PPG-based sensing technologies. Specifically, the aim was (a) to design and develop a pulsatile vascular finger phantom with biorelevant dimensions and mechanical properties; (b) to optically characterize three interchangeable skin color layers and adipose tissue across multiple wavelengths including green, red, and IR; and (c) evaluate the effect of the skin color layers on the PPG signals from the pulsatile finger phantom.

## 2 Methods

### 2.1 Finger Phantom Design

**Fig. 1:**
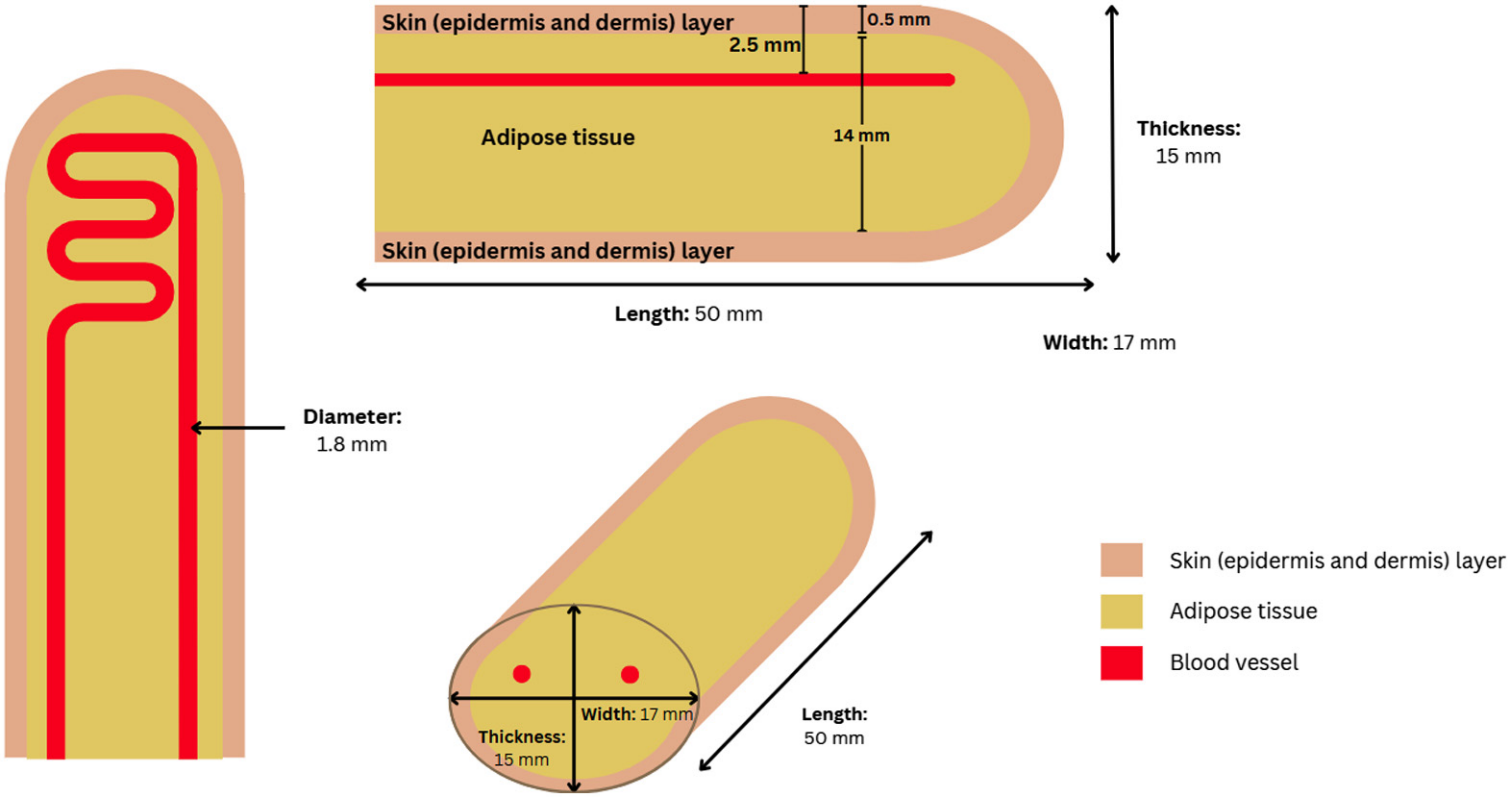
Schematic of the finger phantom with dimensions of 15 mm thickness, 17 mm width, and 50 mm length. The model consists of a skin layer (epidermis and dermis, 0.5 mm thick), an adipose tissue layer (14 mm), and an embedded blood vessel (1.8 mm diameter) positioned 2.5 mm below the top surface.

The finger was chosen as the tissue to simulate because of its widespread use to measure pulse oximetry, one of the biggest applications of the PPG signal. The finger phantom consisted of three components (Fig. 1): a skin layer, adipose or subcutaneous tissue, and an embedded vessel. For the purpose of the study, only the distal and middle phalanges of the finger were modeled, as these are the segments that fit into the pulse oximetry clip. Accurate human finger dimensions are critical for designing a realistic phantom. For practicality reasons, male measurements were selected for the phantom, resulting in a model with dimensions of 18 mm in width, 50 mm in length, and 16 mm in thickness, extending from the fingertip to the base of the middle phalanx, as reported by Tilley.^30^

### 2.2 Custom-Made Silicone Arteries

To simulate dynamic blood volume changes, the phantom included an embedded vasculature system circulating a blood-mimicking fluid. Because fingertip arteries and capillaries measure around 0.8 mm and 6 to 30  μm, respectively,^31^^,^^32^ replicating them is technically challenging due to the lack of commercially available tubing at these scales. To address this limitation, a “zigzag-shaped” alternative vessel arrangement is designed to represent the dense blood perfusion in the fingertip. In addition, replicating vessel biomechanics was essential, as elasticity significantly influences fluid dynamics.^33^ Finger arteries are more muscular than larger vessels, allowing for vasoconstriction and vasodilation to regulate pressure.^34^ To mimic this behaviour, custom vessels were designed with an elastic modulus of 0.7 to 0.8 MPa, corresponding to the longitudinal elasticity as reported in the literature for human finger arteries.^33^^,^^35^ However, circumferential elasticity also plays a key role and varies nonlinearly with pressure due to the structural composition of the arterial wall. The changing arrangement of elastin and collagen fibers results in increasing stiffness with pressure.^34^^,^^36^ At low pressures, elastin dominates, while collagen engagement at higher pressures requires greater force to induce deformation. Consequently, the circumferential elasticity can range from ∼0.3  MPa at low pressures to above 10 MPa at higher pressures.^37^

**Fig. 2:**
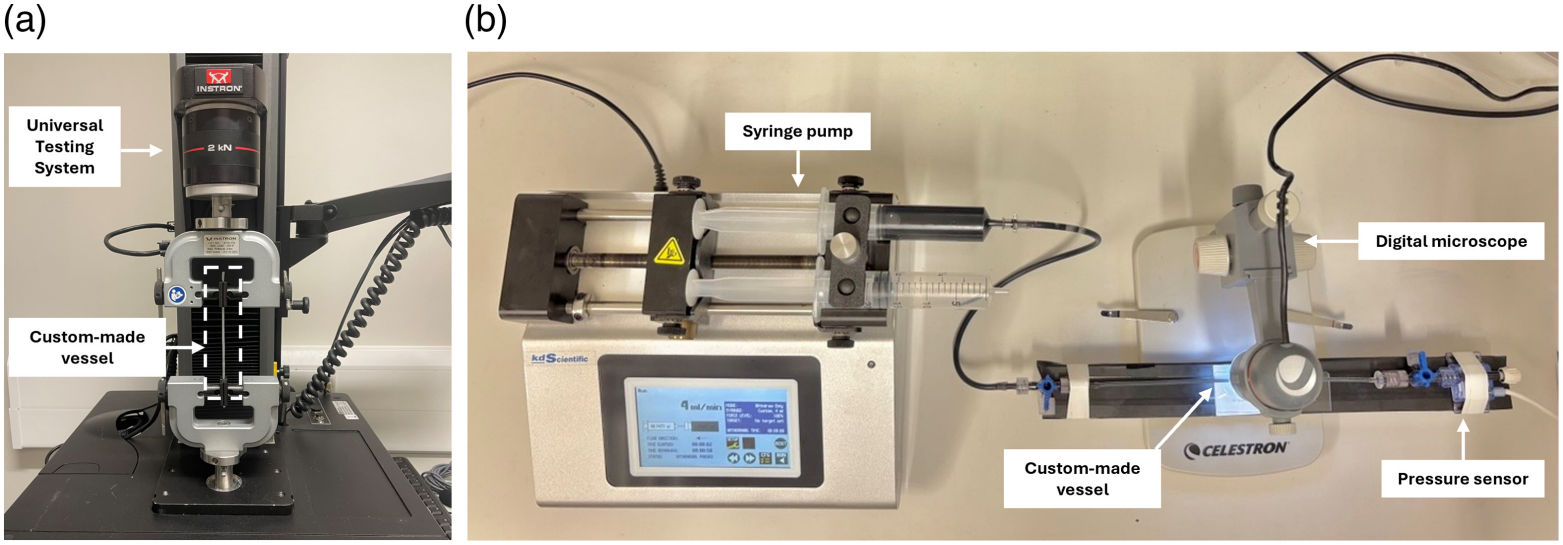
Custom-made vessels mechanical testing. (a) Tensile testing using a Universal Testing System. (b) Compliance measurement setup, where the vessel was filled with diluted India ink and monitored using a pressure sensor.

A common limitation of phantoms with pulsatile flow is the limited expansion of commercially available silicone tubing under systolic pressure, which reduces volume variation and weakens the PPG signal.^23^ To address this, Nomoni et al.^38^ developed a novel technique to fabricate custom vessels that replicate the wall thickness and mechanical properties of real blood vessels. Based on this innovative approach, custom silicone vessels were fabricated. Platsil Gel-00 (Polytek Development Corp., Easton, PA, USA) was used for vessel fabrication. To balance pour time and vessel wall thickness,^39^ the retarder concentration was kept constant at the manufacturer’s recommended maximum of 5%. Hardener (Polytek Development Corp., Easton, PA, USA) was added to modify the vessel’s stiffness. Different vessel recipes with varying ratios of hardener were manufactured. Tensile testing of the custom vessels was performed using a Universal Testing System (Instron 5944, Norwood, MA, USA), with longitudinal Young’s modulus automatically calculated via Bluehill Universal Software (version 4.42, Instron, Norwood, MA, USA) [Fig. 2(a)]. Testing was repeated for six samples to ensure consistency. Wall thickness and outer diameter were measured from cross-sections of the vessels imaged using a handheld digital microscope (Celestron, Torrance, CA, USA) and analyzed with Autodesk Fusion (Autodesk, San Francisco, CA, USA).

The selected custom vessel was evaluated for circumferential compliance, its ability to expand with changing pressure^40^ to ensure it replicates the realistic volumetric expansion of human arteries under pulsatile flow. Defined as the change in cross-sectional area per unit pressure,^26^^,^^33^^,^^41^ compliance was measured by filling the vessel with diluted India ink (Jackson’s Art Supplies, London, UK) and gradually increasing pressure using a syringe pump (Legato 180, KD Scientific, Holliston, MA, USA). Expansion was observed under a handheld digital microscope, whereas a pressure sensor (Broadley-James Ltd., USA) at the opposite end recorded real-time values, as shown in Fig. 2(b).

Circumferential elasticity was estimated from the pressure-diameter relationship obtained in the compliance test. Using the thin-walled cylinder assumption, the circumferential (hoop) stress $\sigma_{\theta }$ and strain $\varepsilon_{\theta }$ were calculated as

$$\sigma_{\theta }=\frac{P\times d}{2t},$$ (1)

and

$$\varepsilon_{\theta }=\frac{\Delta D}{D_{0}},$$ (2)

where P is the applied pressure, d is the inner diameter, t is the wall thickness, ΔD is the change in diameter, and $D_{0}$ is the baseline diameter.

### 2.3 Mechanical Characterization

In this study, the two-part silicone Platsil Gel-00 elastomer was chosen within the room-temperature-vulcanizing silicones based on its availability, ease of handling, and prior experience indicating improved stability of incorporated optical absorbers in this specific silicone.

Reported values for the hardness from the fingertip were considered to guide material selection. As shown in Table 1, fingertip hardness ranged from 25.7 to 35.0 (Shore OO).^23^^,^^42^

**Table 1:** Finger tissue hardness from literature.^23^^,^^42^

Anatomical location (measurement instrument)	Hardness	Author, year
Distal phalanx of the index finger (Rex Model 1700 Max-Hand Durometer, Type 00)	32.0 ± 3.0 (Shore OO)	(Falanga and Bucalo, 1993)^42^
Right index finger (AD-100-OO Precision Shore Durometer)	29.4 ± 3.7 (Shore OO)	(Nomoni et al., 2020)^23^

Hardness measurements were performed using the AD-100-OO Precision Shore Durometer (Checkline Europe, Enschede, The Netherlands), which is commonly used for soft materials such as animal tissue. The testing procedure followed the requirements outlined in ASTM D2240-15.^43^ According to the standard, the test specimen must be at least 6.0 mm thick, and the hardness value should be calculated as the mean or median of five measurements, each taken at least 12.0 mm from any edge and no less than 6.0 mm apart.^43^ The durometer was placed on the surface of the tissue with the footpad positioned at a slight angle and then gently rolled into a flat position to ensure the most accurate reading.

### 2.4 Optical Characterization

Understanding and accurately replicating the optical properties of biological tissues is essential for developing realistic tissue phantoms for light-tissue interaction studies. In this work, the optical properties of a silicone-based phantom were modified to mimic those of adipose tissue and three distinct skin tone groups: pale, medium, and dark.

#### 2.4.1 Adipose tissue

For the adipose tissue, the target value range was determined based on a literature search of reported spectra of absorption ($\mu_{a}$) and reduced scattering coefficients ($\mu_{s}^{\prime }$) of human adipose tissue.^44^^–^^48^ Only studies covering the range of primary interest for this study: from 500 to 1000 nm wavelengths were included. A summary of the selected studies is presented in Table S1 in the Supplementary Material, detailing their measurement sites, techniques, instrumentation, sample characteristics, wavelength coverage, study objectives, blood content status, sample preparation methods, and assumed anisotropy factors. This comparison also highlights the methodological variability that may impact the consistency and comparability of optical property data across studies.

The optical properties of the adipose phantom were tailored by incorporating varying concentrations of scatterers and absorbers. Titanium dioxide (${\mathrm{TiO}}_{2}$; Kronos Worldwide Inc., Dallas, TX, USA) was used as the scatterer, whereas Bismarck Brown (BDH Chemicals Ltd., London, England) and India Ink were implemented as the absorbers. The formulation that best matched the reported optical properties was developed through the following process. A stock solution of Bismarck Brown was prepared in isopropanol at a concentration of 25  mg/mL, from which 8.57  μL per gram of silicone mix was added. In addition, 7.14  μL per gram of diluted India Ink (1% v/v in deionized water) was incorporated. ${\mathrm{TiO}}_{2}$ was added at 1 mg per gram of silicone mix. These additives were thoroughly stirred into silicone part A to ensure a uniform mix. A 1.5% concentration of retarder was then added to control the curing rate, followed by mixing with silicone part B. The resulting mixture was then degassed in a vacuum chamber for 3 min to eliminate air bubbles.

#### 2.4.2 Skin color layers

For the skin layers, specific optical properties were targeted to simulate pale, medium, and dark skin tones. Target ranges were selected according to the literature, with optical properties of at least two skin color groups, using the Fitzpatrick scale or ethnicity, as these were the most common skin classification methods.^47^^,^^49^^,^^50^ A summary of the selected studies is presented in Table S2 in the Supplementary Material, outlining details on study type, measurement sites and techniques, instrumentation, sample characteristics, wavelength range, and study objectives. Although skin optical properties have been widely studied, many reports lack a clear classification of skin tone, and values vary due to differences in experimental methods. Still, a consistent trend is observed: absorption increases with melanin content, especially in the visible range, with differences diminishing in the IR.^7^ By contrast, reduced scattering coefficients show no clear pattern, likely due to variations in hydration, texture, and other physiological factors.^7^

To develop skin phantoms representative of different skin tones, the absorption coefficient ($\mu_{a}$) was tuned to match values reported for pale, medium, and dark skin. This was achieved by adjusting the concentrations of scatterers and absorbers. ${\mathrm{TiO}}_{2}$ served as the scattering agent, whereas Bismarck Brown, India Ink, and Epolight dye 5532 (Epolin Inc., Newark, New Jersey, USA) were used as absorbers. Three formulations were identified that closely replicated the target optical properties. The same stock solution of Bismarck Brown (25  mg/mL in isopropanol) used for the adipose phantom was used here, along with a new stock solution of Epolight 5532 (1  mg/mL in acetone). Per gram of silicone, the absorber concentrations were as follows:

• Pale skin: 20  μL Bismarck Brown, 70  μL Epolight 5532, and 10  μL India Ink (1% v/v)
• Medium skin: 40  μL Bismarck Brown, 100  μL Epolight 5532, and 20  μL India Ink (1% v/v)
• Dark skin: 130  μL Bismarck Brown, 400  μL Epolight 5532, and 30  μL India Ink (1% v/v)

${\mathrm{TiO}}_{2}$ was added at 1  mg/g of silicone in all formulations. Additives were thoroughly mixed into silicone part A, followed by the addition of 1.5% retarder to control the curing rate. The mixture was then combined with part B, degassed for 3 min in a vacuum chamber to remove air bubbles.

#### 2.4.3 Determination of $\mu_{a}$ and $\mu_{s}^{\prime }$

**Fig. 3:**
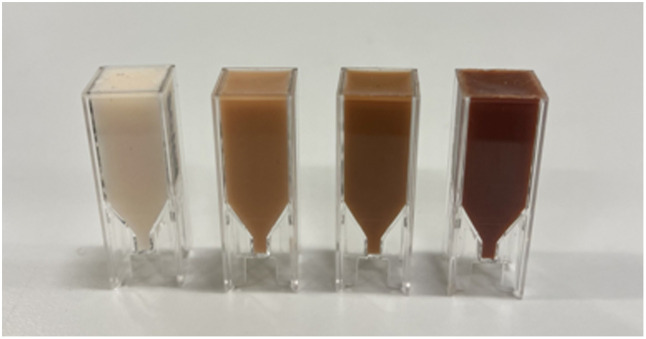
Adipose, pale, medium, and dark skin samples (left to right) used for optical properties measurement.

Total transmittance and reflectance were measured from the adipose and the three skin colour layers (Fig. 3) using a Lambda 1050 dual-beam spectrophotometer equipped with a 100-mm integrating sphere and a PMT/InGaAs detector (Perkin Elmer Corp., Waltham, MA, USA). These values were then used to estimate the $\mu_{a}$ and $\mu_{s}^{\prime }$ through the Inverse-Adding Doubling (IAD) method. The calculations assumed an anisotropy factor of 0.9^44^^,^^47^^,^^51^^,^^52^ and refractive index of 1.4 for the silicone, which closely aligns with that of soft tissue (1.33 to 1.50).^24^^,^^53^ To ensure consistency, five samples were prepared and measured from each phantom (adipose, pale, medium, and dark skin). Each sample’s spectral measurements were repeated three times and averaged. Samples were prepared on different days to assess reproducibility.

### 2.5 Finger Phantom Fabrication

**Fig. 4:**
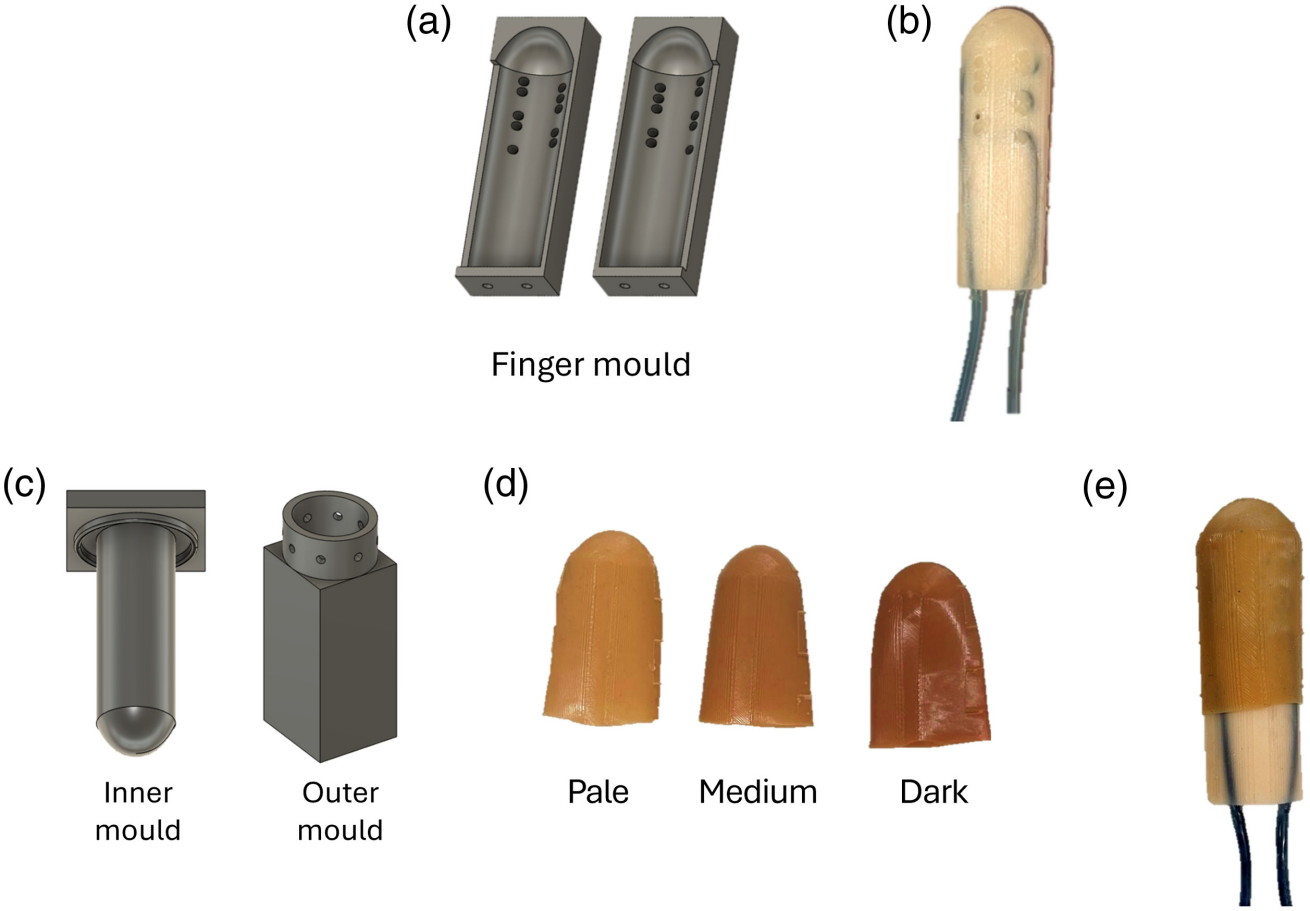
Finger phantom process. (a) Two-part adipose finger mould. (b) Adipose finger phantom after curing and mould removal. (c) Two-part skin “finger glove” mould system for producing the 0.5-mm-thick silicone skin “finger glove,” consisting of an inner mould shaped similar to the adipose phantom and an outer mould defining the external finger geometry. (d) Pale, medium, and dark skin color “finger gloves.” (e) Final finger phantom with a skin “finger glove” fitted over the adipose tissue.

A two-part 3D-printed mould was used to fabricate the finger phantom [Fig. 4(a)]. The process began by placing the custom vessel inside the mould, followed by pouring a silicone mixture to simulate adipose tissue. Wire strands temporarily supported the vessel during curing and were removed afterward, leaving holes that were filled with additional silicone. Once cured, the mould was removed to reveal the adipose finger phantom [Fig. 4(b)]. To create the skin layer, a “finger glove” was moulded using a two-part system: an inner mould shaped similar to the adipose phantom and an outer mould representing the negative of the final finger shape [Fig. 4(c)]. Silicone was poured into the outer mould, and the inner mould was inserted, creating a 0.5-mm gap for the skin layer. Escape holes allowed excess silicone to exit, ensuring uniform thickness. After curing, the inner mould was removed, yielding a 0.5-mm-thick silicone glove that fit precisely over the adipose phantom. Figure 4(d) shows the final skin gloves in pale, medium, and dark tones, and finally, the finger phantom with the skin “finger glove” is shown in Fig. 4(e).

### 2.6 *In Vitro* Cardiovascular System Configuration

**Fig. 5:**
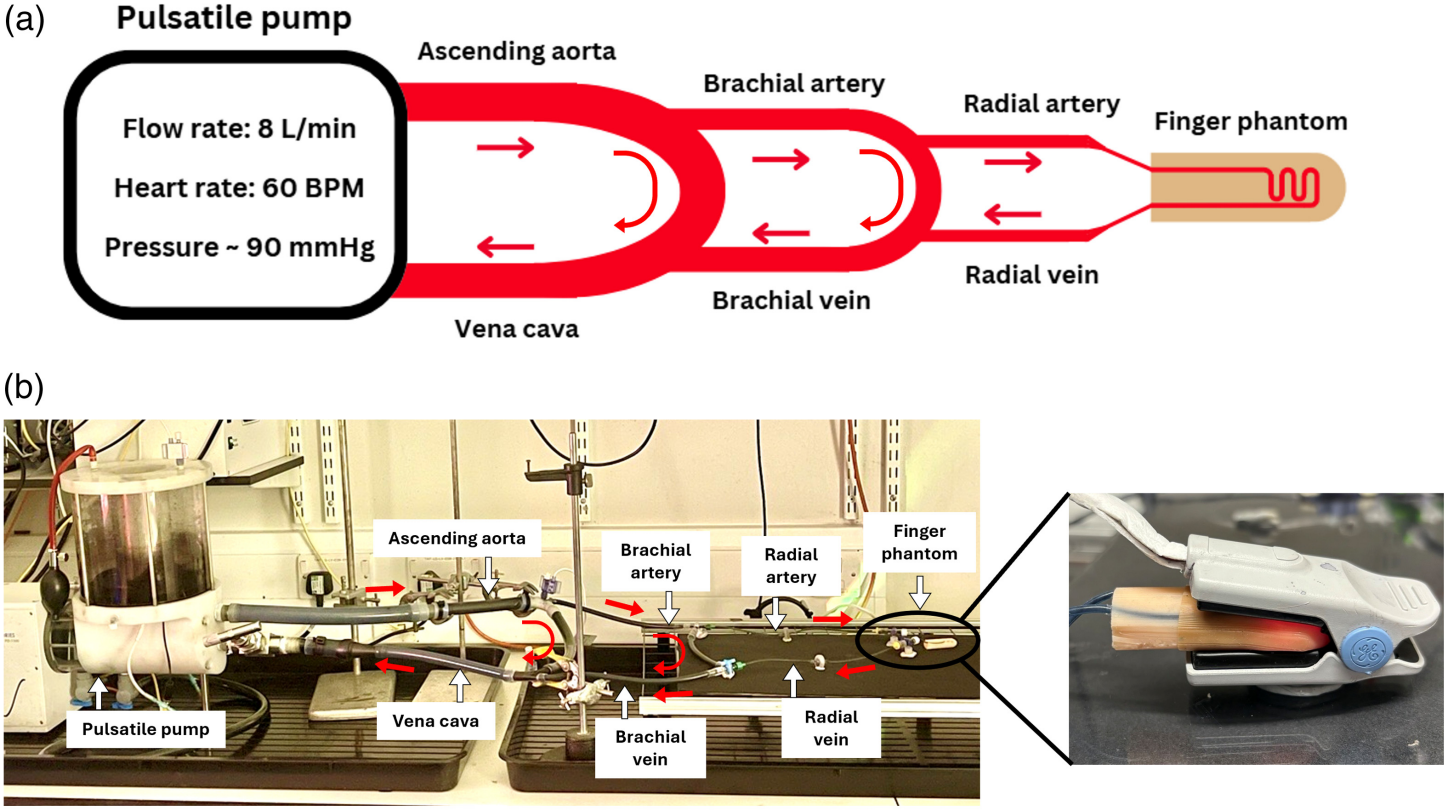
Pump setup. (a) The system comprises a computer-controlled pulsatile pump, tubing, and a vascular finger phantom. Pump settings were a flow rate of 8  L/min, a heart rate of 60 bpm, and a mean pressure of ∼90  mmHg. (b) *In vitro* setup based on the schematic, with a zoomed-in image (right) of the finger phantom inside the finger clip probe.

The finger phantom was integrated into an *in vitro* cardiovascular system, a closed-loop pulsatile system to replicate physiological conditions of the upper body. A PD-1100 Pulsatile Pump (BDC Labs, USA), driven by a computer-controlled linear motor, generated flow at 60 bpm, 8  L/min flow rate, and ∼90  mmHg system pressure. To isolate the motor from fluid contact, a fluid isolation module was used. The flow pathway mimicked the human circulatory system using PVC tubing: from the “heart” through the ascending aorta (internal diameter = 20 mm),^54^^,^^55^ brachial artery (internal diameter = 5.3 mm),^56^^,^^57^ and radial artery (internal diameter = 2.3 mm),^57^^,^^58^ into the vascular finger phantom (Fig. 5). The fluid exited through the radial and brachial veins, returning to the superior vena cava. A resistance clamp simulated peripheral resistance.

A custom-made PPG finger clip probe operating in reflectance mode was used to acquire signals at three wavelengths: green (530 nm), red (655 nm), and IR light (940 nm) as shown in Fig. 5. The distance between the LEDs and the photodetector was 4 mm. The PPG finger clip probe was interfaced with a PPG acquisition system developed by the Research Center for Biomedical Engineering (RCBE),^59^ at a sampling rate of 2 kHz. Each LED was driven with a current of 40 mA.

To approximate the optical absorption properties of arterial blood, a blood-mimicking fluid was prepared using a dye. It consisted of 2 mL of India ink, 700 mg of Wright stain (Thermo Fisher Scientific Inc, Waltham, MA, USA), and 400 mg of Congo red (BDH Chemicals LTD, Poole, UK) in 1 L of deionized water. This composition serves as the standard recipe for preparing 1 L of the blood–mimicking fluid with an absorption level enough for the system to produce PPG signals across 500 to 1000 nm.

### 2.7 Feature Extraction and Statistical Analysis

Raw PPG data were extracted from the acquisition system using a text file and processed in MATLAB (version 2022a). The AC component of the signal was obtained by filtering with a fourth-order Butterworth bandpass with cutoff frequencies of 0.4 and 10 Hz. The DC component was obtained using a fourth-order Butterworth low pass with a 0.3 Hz cutoff frequency.

To evaluate the influence of skin tone on PPG signal characteristics, four features were analyzed: signal-to-noise ratio (SNR), peak-to-peak amplitude, area under the curve (AUC), and AC/DC ratio, across three skin tones: pale, medium, and dark. Peak-to-peak amplitude was calculated as the difference between the detected peaks and troughs in the AC signal on a per-beat basis. The AUC was also computed per beat using the MATLAB function. SNR was calculated using MATLAB function and was evaluated in 10-s windows, with noise obtained using a fourth-order Butterworth high-pass filter with a cutoff frequency of 10 Hz. The AC/DC ratio was calculated by dividing the AC component by the average value of the DC component for each detected pulse and then multiplying by 100 to express the results as a percentage. As skin tone darkens, reduced signal amplitude and increased noise are expected.

Statistical analysis began with a Shapiro-Wilk test for normality. Due to non-normal distributions in several groups, non-parametric methods were used. A Kruskal-Wallis test assessed overall differences for each feature, followed by Dunn-Sidak post hoc tests for pairwise comparisons. Mean rank differences were calculated to quantify effect magnitude between skin tone groups, and effect size was estimated using eta squared ($\eta^{2}$), indicating the proportion of variance explained by skin tone.

## 3 Results

### 3.1 Vessel Manufacture

An optimal longitudinal elastic modulus of 0.79±0.03  MPa, which closely matched the literature values of 0.7 to 0.8 MPa, was achieved using Platsil Gel-00 with 5% retarder and 18% hardener. The resulting dimensions were a wall thickness of 0.27±0.01  mm and an outer diameter of 1.81±0.04  mm, and these are detailed in Table 2.

**Table 2:** Thickness, inner diameter, outer diameter, and longitudinal elasticity for custom-made vessels fabricated using Platsil Gel-00 with 5% retarder and 18% hardener.

Wall thickness (mm)	Inner diameter (mm)	Outer diameter (mm)	Longitudinal elasticity (MPa)
0.27 ± 0.01	1.26 ± 0.04	1.81 ± 0.04	0.79 ± 0.03

**Fig. 6:**
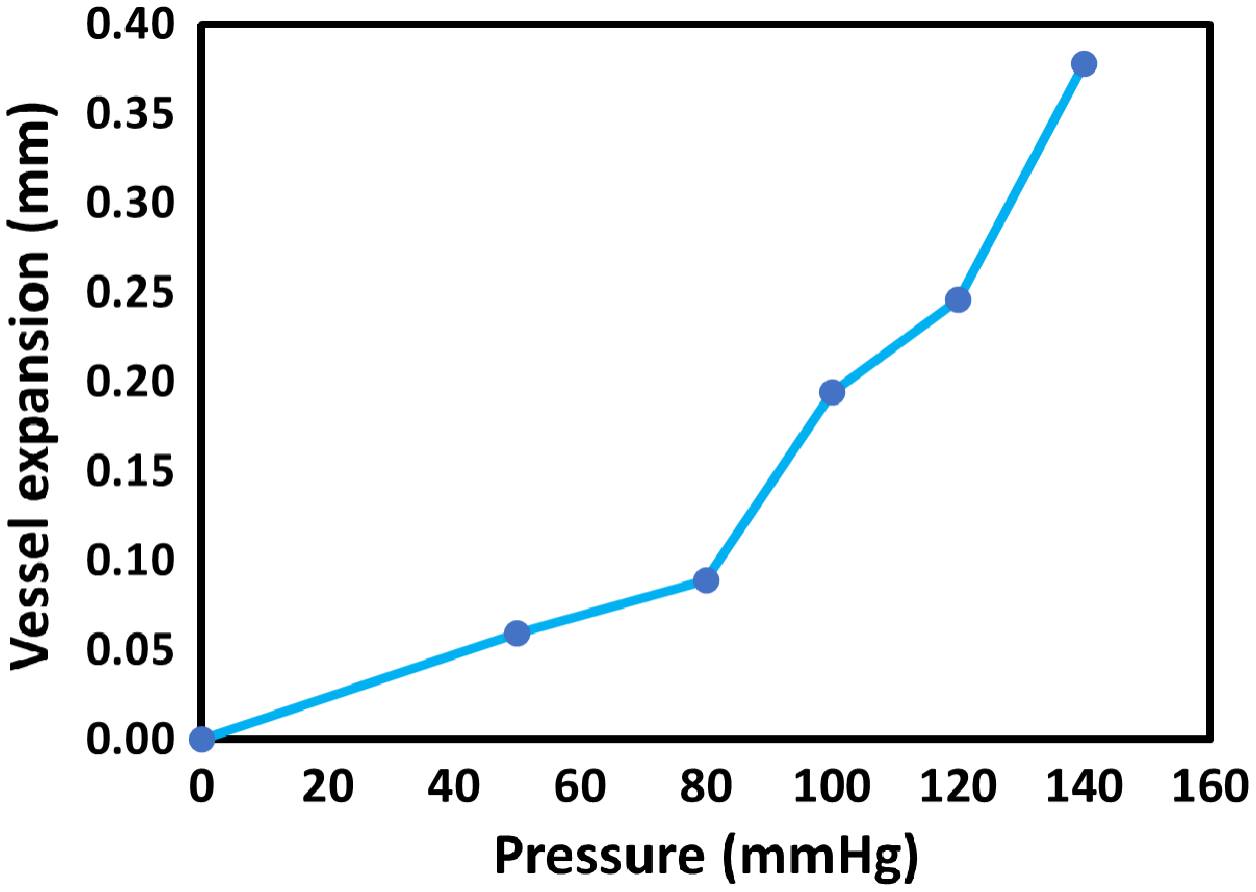
Compliance test of the custom vessel. Vessel expansion (mm) is plotted against applied pressure (mmHg). Dark blue points indicate the measured values relative to the baseline (0 mmHg), and the light blue line connects the points to show the overall trend. Only a single measurement was performed at each pressure level; therefore, error bars are not included.

The results of the compliance test are shown in Fig. 6. The dark blue points represent the measured vessel expansion (mm), calculated relative to the baseline (0 mmHg). As pressure increased, the vessel progressively expanded. The light blue line connects the data points to illustrate the overall trend.

The circumferential elasticity ($\sigma_{\theta }/\varepsilon_{\theta }$), calculated from the pressure-diameter relationship, ranged approximately from 0.21 to 0.50 MPa between 50 and 140 mmHg. A 20% change in the diameter of the lumen was achieved by a pressure difference of 140 mmHg, crucial for PPG imaging systems.^41^ Table 3 summarizes the vessel diameter expansion at increasing pressure levels. The baseline lumen diameter of the custom vessel was 1.81 mm. Expansion was measured relative to this baseline, and the percentage change was calculated as the ratio of the measured expansion at each pressure to the baseline diameter of the vessel, expressed as a percentage. The largest change occurred at 140 mmHg, corresponding to a 0.38 mm increase in diameter, or ∼20% of the baseline value. This nonlinear response reflects the vessel’s increasing compliance under higher pressures.

**Table 3:** Measured diameter expansion and calculated circumferential elasticity of the custom-made vessel at increasing pressures.

Pressure (mmHg)	Vessel expansion (mm)	Vessel expansion percentage change (%)	Circumferential elasticity (MPa)
50	0.06	3.14	0.47
80	0.09	4.74	0.50
100	0.19	10.33	0.30
120	0.25	13.10	0.27
140	0.38	20.13	0.21

### 3.2 Mechanical Characterization

**Fig. 7:**
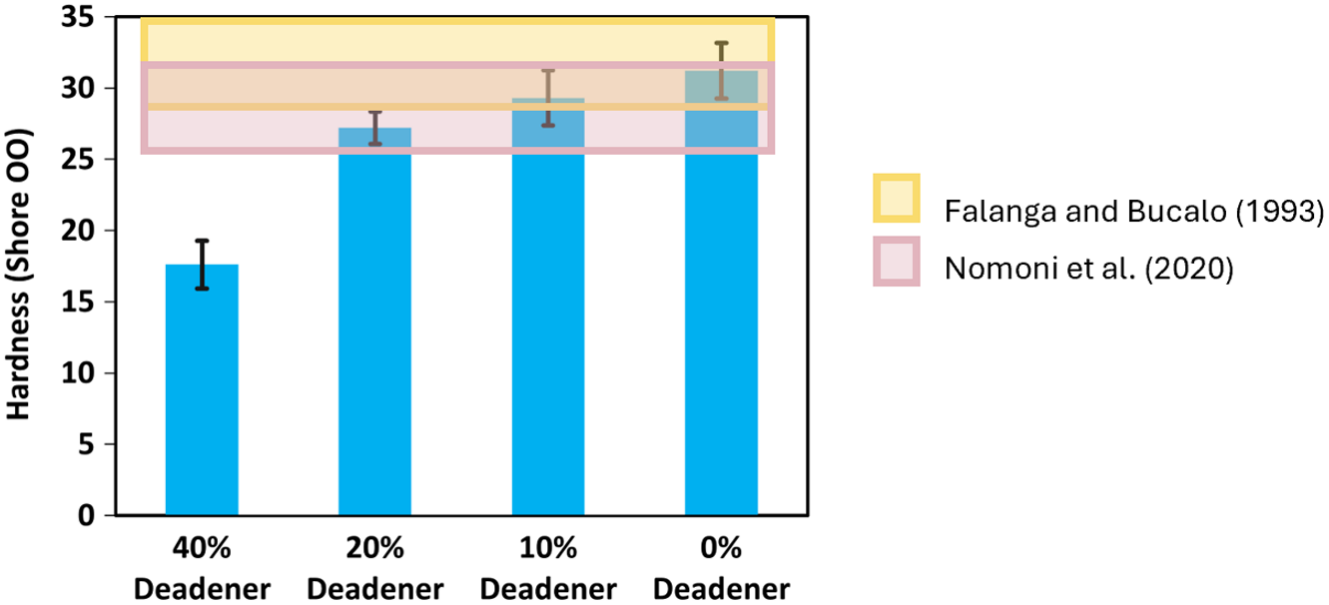
Measured hardness of silicone samples with varying deadener content (0%, 10%, 20%, and 40%). Bars represent mean hardness values (Shore OO) with error bars indicating standard deviations. Shaded areas denote reported hardness ranges for human finger tissue: pink (Nomoni et al.^23^) and yellow (Falanga and Bucalo^42^).

Values reported in literature indicated that the hardness of the finger tissue falls within a range of 25.7 to 35.0 (Shore OO), as reported by Nomoni et al.^23^ and Falanga and Bucalo.^42^ This range is illustrated in Fig. 7 as shaded reference areas. In comparison, the mean measured hardness of the tested silicone samples ranged from 17.6 to 31.2 (Shore OO), with lower values corresponding to higher deadener content, as shown in Fig. 7.

The silicone mixture that best matched the literature range was the formulation without deadener, which achieved a biorelevant hardness of 31.2±1.9 (Shore OO). This result was expected, as the manufacturer’s technical bulletin for Platsil Gel 00 reports a cured hardness of Shore OO–30.^60^

### 3.3 Optical Characterization

**Fig. 8:**
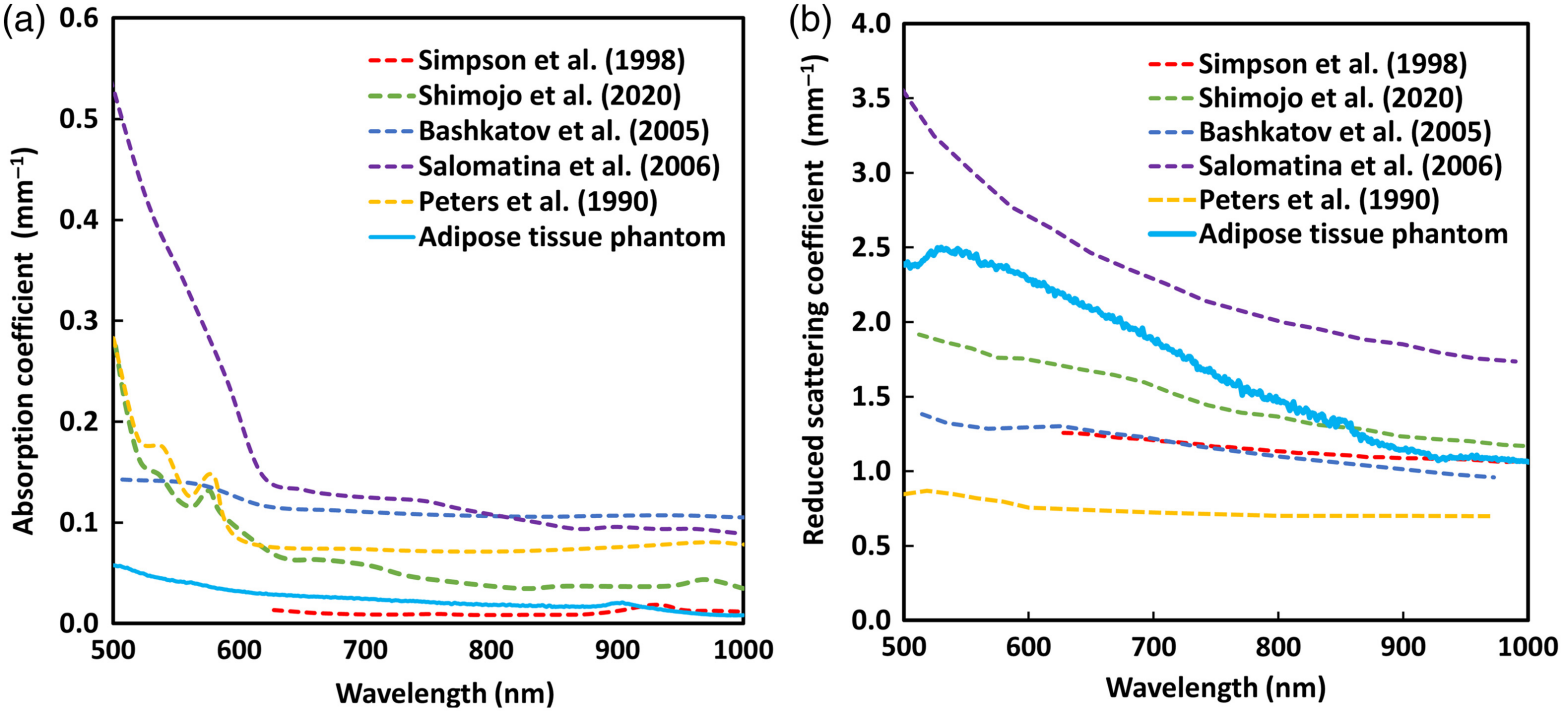
Optical properties of adipose finger phantom. (a) Mean absorption coefficient and (b) mean reduced scattering coefficient spectra within the range of 500 to 1000 nm, compared with reported values for human adipose tissue from the literature.^44^^–^^48^

The absorption and reduced scattering coefficients of the resulting adipose phantom tissue are shown in Fig. 8, where they are compared with those reported in the literature.^44^^–^^48^

**Fig. 9:**
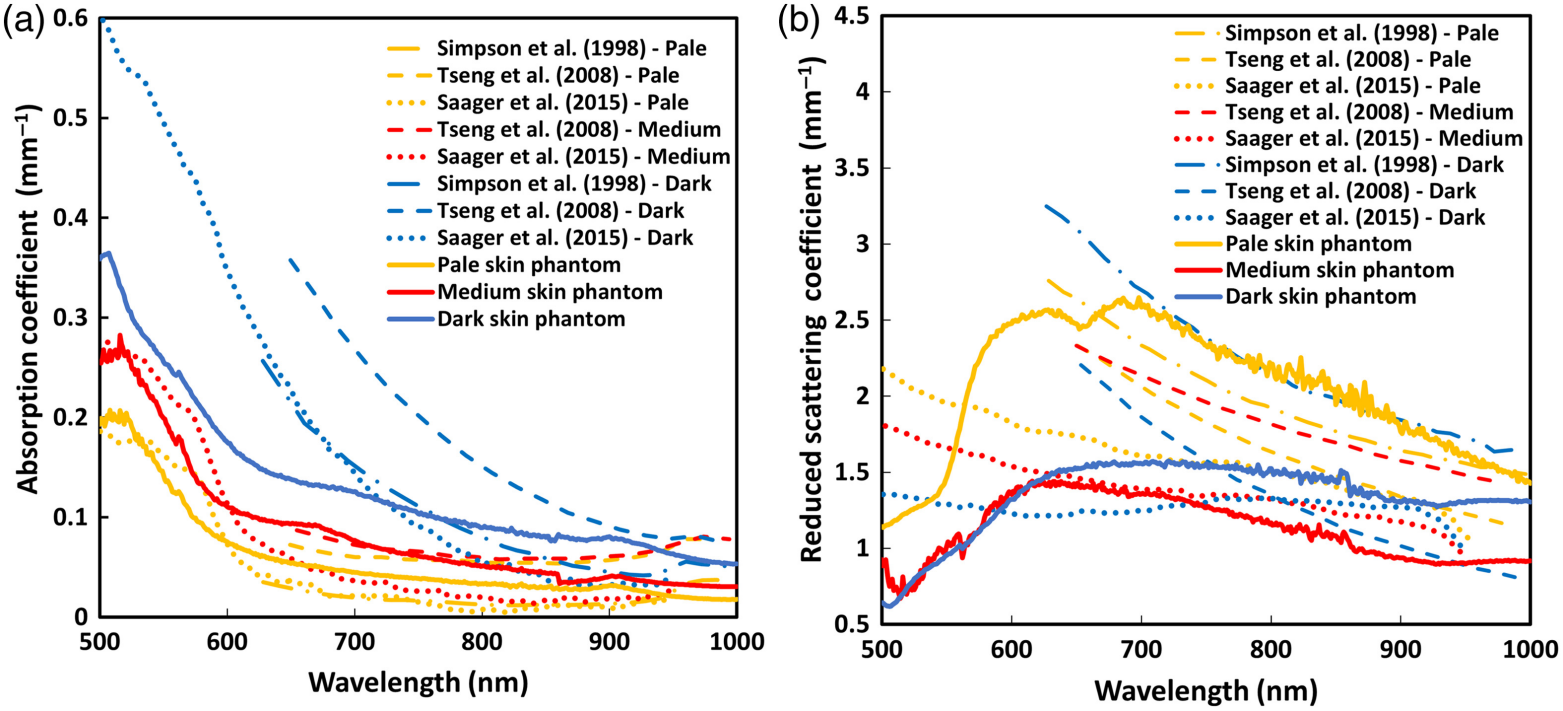
Optical properties of pale, medium, and dark skin tones. (a) Mean absorption and (b) mean reduced scattering coefficient spectra within the range of 500 to 1000 nm, compared with reported values of human skin from the literature.^47^^,^^49^^,^^50^

The absorption and reduced scattering coefficients for the three skin color phantoms are shown in Fig. 9, where they are compared to the values found in the literature.^47^^,^^49^^,^^50^

As with the adipose tissue phantom, the optical characterization of the skin color phantoms focuses on three specific wavelengths: 530, 655, and 940 nm, which are commonly used in wearable devices. The absorption and reduced scattering coefficients at these wavelengths are summarized in Table 4.

**Table 4:** Absorption ($\mu_{a}$) and reduced scattering coefficients ($\mu_{s}^{\prime }$) of the developed adipose tissue and skin color phantoms at 530, 655, and 940 nm.

Wavelength [nm]	$\mu_{a}$ $\left[{\mathrm{mm}}^{-1}\right]$				$\mu_{s}^{\prime }$ $\left[{\mathrm{mm}}^{-1}\right]$			
	Adipose tissue	Pale skin	Medium skin	Dark skin	Adipose tissue	Pale skin	Medium skin	Dark skin
530	0.047	0.183	0.248	0.290	2.500	1.278	0.854	0.835
655	0.027	0.053	0.092	0.134	2.088	2.462	1.403	1.529
940	0.013	0.022	0.034	0.067	1.091	1.642	0.902	1.291

### 3.4 PPG Waveforms

**Fig. 10:**
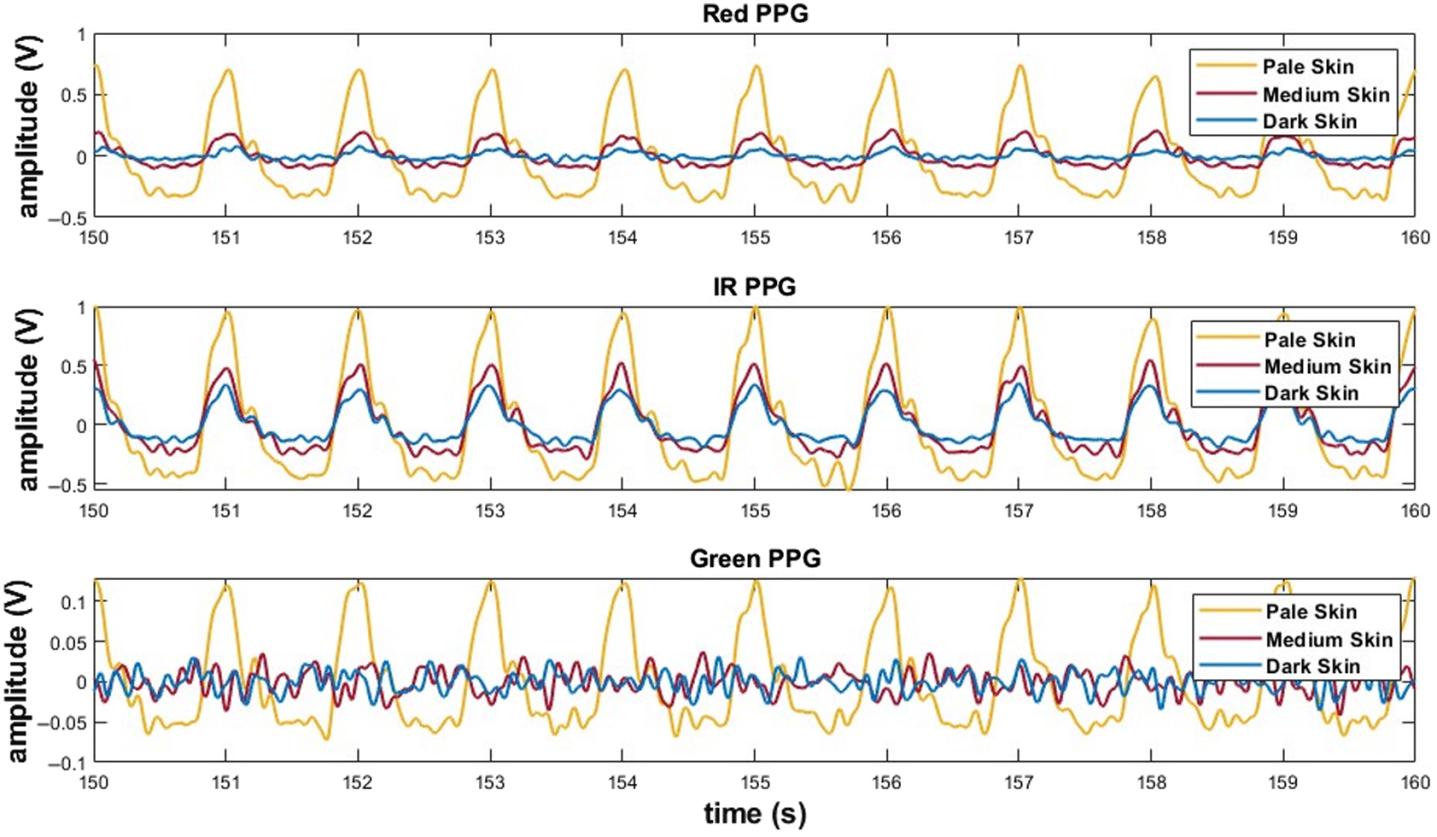
10-s window for red, infrared (IR), and green PPG signals in reflectance mode from the finger phantom with pale (yellow), medium (red), and dark (blue) skin.

The *in vitro* cardiovascular system described in Sec. 2.6 was used to generate a pulsatile flow through the phantom, simulating physiological volume changes. The system operated at 60 bpm, with an 8  L/min flow rate and an approximate pressure of 90 mmHg. The custom finger clip probe acquired reflectance-mode PPG signals at 530, 655, and 940 nm. Signals were recorded for 5 min in a dark room. A 10-s window of the filtered PPG signals is shown in Fig. 10.

### 3.5 Extracted Features and Statistical Analysis

**Fig. 11:**
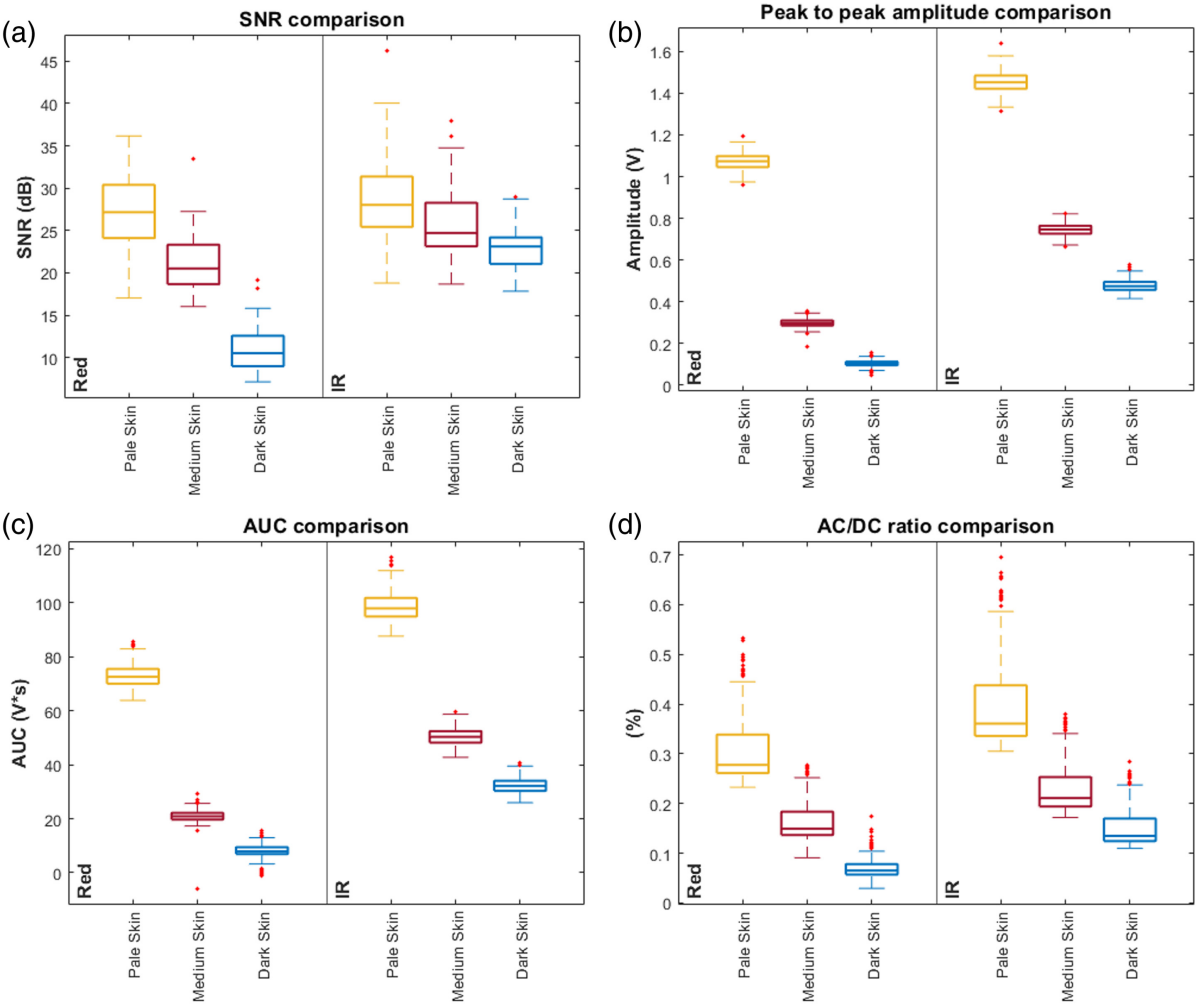
Boxplot comparison of key signal features. (a) Signal-to-noise ratio (SNR), (b) peak-to-peak amplitude, (c) area under the curve (AUC), and (d) AC/DC ratio box plot comparison. Illustrated for pale (yellow), medium (red), and dark (blue) skin, across red and infrared (IR) wavelengths.

SNR, peak-to-peak amplitude, AUC, and AC/DC ratio were extracted from the PPG signals across all skin tones and are shown in box plots comparing their distribution across different skin tones (Fig. 11). In addition, Table 5 presents the median and interquartile range (IQR) values for each feature at red and IR wavelengths (655 and 940 nm), along with the corresponding sample sizes used in the analysis. These provide a summary of the central tendency and variability observed in the data for each skin tone condition.

**Table 5:** Median and interquartile ranges (IQR) for peak-to-peak amplitude, signal-to-noise ratio (SNR), area under the curve (AUC), and AC/DC ratio across red and infrared (IR) wavelengths for each skin tone condition. Corresponding sample sizes (n) are also reported for each feature.

Skin	Red (655 nm)		IR (940 nm)	
	Median	IQR	Median	IQR
SNR (dB), n=59				
Pale	27.159	6.281	28.030	5.939
Medium	20.520	4.651	24.707	5.145
Dark	10.522	3.591	23.111	3.128
Amplitude peak-to-peak (V), n=298				
Pale	1.074	0.053	1.452	0.064
Medium	0.297	0.024	0.747	0.038
Dark	0.103	0.018	0.475	0.038
AUC (V*s), n=297				
Pale	72.533	5.453	97.901	6.877
Medium	20.899	2.465	50.324	4.236
Dark	7.940	2.575	32.164	3.729
AC/DC ratio (%), n=297				
Pale	0.278	0.078	0.361	0.102
Medium	0.149	0.047	0.211	0.059
Dark	0.065	0.021	0.135	0.045

Although signals were initially recorded for red, IR, and green wavelengths, PPG signals acquired with the green wavelength were only distinguishable in the pale skin condition. For medium and dark skin tones, the green PPG signals did not contain discernible pulsatile waveforms and consisted primarily of noise, making beat detection impossible. As a result, statistical analysis was restricted to the red (655 nm) and IR (940 nm) wavelengths, where PPG signals were obtained across all skin tones.

Statistical comparisons between skin tone groups were conducted using the Kruskal-Wallis test followed by Dunn-Sidak post hoc pairwise comparisons. Table 6 presents the mean rank differences, p values, and effect sizes ($\eta^{2}$) for each pair of skin tones across the red and IR wavelengths (655 and 940 nm), based on four PPG features: SNR, peak-to-peak amplitude, AUC, and AC/DC ratio. The mean rank difference represents how far apart the distributions of two groups are, and higher values indicate larger differences. The effect size ($\eta^{2}$) quantifies the magnitude of these differences, and values closer to 1 suggest that a greater proportion of the variability is explained by skin tone.

**Table 6:** Pairwise comparisons of PPG signal features between skin tone group (pale, medium, dark) across red (655 nm), and infrared (940 nm) wavelengths. Reported metrics include mean rank differences, p values (Dunn-Sidak), and effect sizes ($\eta^{2}$) for signal-to-noise ratio (SNR), amplitude peak-to-peak, area under the curve (AUC), and AC/DC ratio. Statistically significant differences (p<0.05) are indicated with an asterisk *.

Skin 1	Skin 2	Red (655 nm)			IR (940 nm)		
		Mean rank difference	p value	$\eta^{2}$	Mean rank difference	p value	$\eta^{2}$
SNR (dB)							
Pale	Medium	41.780	*p* < 0.001*	0.759	22.542	0.050	0.197
Pale	Dark	108.576	*p* < 0.001*		56.627	*p* < 0.001*	
Medium	Dark	66.797	*p* < 0.001*		34.085	*p* <0.001*	
Amplitude peak-to-peak (V)							
Pale	Medium	298.000	*p* < 0.001*	0.885	298.000	*p* < 0.001*	0.888
Pale	Dark	596.000	*p* < 0.001*		596.000	*p* < 0.001*	
Medium	Dark	298.000	*p* < 0.001*		298.000	*p* < 0.001*	
AUC (V*s)							
Pale	Medium	298.003	*p* < 0.001*	0.885	297.000	*p* < 0.001*	0.888
Pale	Dark	592.997	*p* < 0.001*		594.000	*p* < 0.001*	
Medium	Dark	294.993	*p* < 0.001*		297.000	*p* < 0.001*	
AC/DC ratio (%)							
Pale	Medium	290.347	*p* < 0.001*	0.868	307.347	*p* < 0.001*	0.777
Pale	Dark	587.421	*p* < 0.001*		554.906	*p* < 0.001*	
Medium	Dark	297.074	*p* < 0.001*		247.559	*p* < 0.001*	

For the SNR, the strongest differences were observed at 655 nm, particularly between pale and dark skin tones, with a mean rank difference of 108.576 and a large effect size ($\eta^{2}=0.759$), indicating that 75.9% of the variance in SNR at this wavelength can be attributed to differences in skin tone. Moderate differences were also seen between medium and dark skin (Δrank=66.797), with all comparisons at this wavelength reaching statistical significance. At 940 nm (IR), the SNR differences were smaller, but still significant between pale and dark skin (Δrank=56.627, $\eta^{2}=0.197$), suggesting IR light is less influenced by pigmentation.

For peak-to-peak amplitude, all pairwise comparisons were statistically significant across both wavelengths, with consistently large differences. At 655 nm, the largest observed rank difference was between pale and dark skin (Δrank=596.000, $\eta^{2}=0.885$), and the same difference at 940 nm (Δrank=596.000, $\eta^{2}=0.888$). These results show that amplitude is highly sensitive to skin pigmentation across both wavelengths.

A similar trend was observed for AUC, with all pairwise comparisons yielding significant differences. The most substantial contrasts were again between pale and dark skin tones, with similar rank differences at both wavelengths (Δrank=592.997 at 655 nm, and Δrank=594.000 at 940 nm), each associated with a large effect size ($\eta^{2}=0.885$ and 0.888, respectively). This indicates that ∼88% of the variability in AUC can be explained by skin tone.

For the AC/DC ratio, the trend continued, with large rank differences and high effect sizes across all comparisons. The greatest difference was between pale and dark skin at 655 nm (Δrank=587.421, $\eta^{2}=0.868$) and at 940 nm (Δrank=554.906, $\eta^{2}=0.777$). These results confirm that the AC/DC ratio is also strongly affected by skin pigmentation, although slightly less in the IR light.

Overall, red light (655 nm) showed the strongest difference between skin tones across all features, whereas IR light (940 nm) exhibited reduced but still significant effects. This effect was strongest in amplitude-related features and most evident between the pale and dark skin tones.

## 4 Discussion

Light-based sensing technologies such as PPG are widely used in clinical and wearable health monitoring devices.^5^^,^^22^ However, disparities in signal quality across different skin tones have raised important concerns regarding bias and inclusivity in physiological monitoring.^6^ It has been stated that variations in melanin concentration significantly affect how light is absorbed and scattered in the skin, especially in the visible range.^7^^,^^47^ These differences can reduce measurement accuracy and reliability in darker skin tones, and from an engineering perspective, understanding these disparities requires a deeper exploration of light-tissue interactions. The purpose of this study was to address this need by developing a pulsatile vascular finger phantom with realistic anatomical, mechanical, and optical properties to evaluate the impact of skin pigmentation on the PPG signal.

A key advantage of this work is the development of custom-made silicone vessels, fabricated using the methodology from Nomoni et al.^23^ Unlike commercially available tubing, which often exhibits minimal expansion under systolic pressure,^23^ these vessels were designed to mimic the longitudinal elasticity and compliance of human finger arteries. Mechanical characterization confirmed that the fabricated vessels achieved a longitudinal Young modulus of 0.79±0.03  MPa, closely matching the literature-reported range of 0.7 to 0.8 MPa^33^^,^^35^ for human finger arteries. In addition, circumferential elasticity was evaluated from the pressure-diameter relationship, yielding values between 0.21 and 0.50 MPa over the physiological pressure range 50 to 140 mmHg. These results are consistent with the nonlinear mechanical behaviour of human arteries,^37^ where the circumferential elasticity increases from ∼0.3  MPa at low pressures to above 10 MPa at higher pressures due to collagen engagement.^37^ Blood pressure produces both circumferential and longitudinal stresses in the arterial wall. Accurately replicating these coupled responses is essential for realistic pulsatile vessel expansion and consequently PPG signal formation.

Although the custom silicone vessels exhibited a slightly cloudy appearance due to the intrinsic properties of Platsil Gel-00, this did not affect their optical performance. Spectrophotometer measurements of the cured silicone confirmed that neither the absorption nor scattering was significant across the tested wavelengths (530, 655, and 940 nm). Therefore, the vessel translucency does not influence the measured PPG signal intensity or light attenuation.

The mechanical realism of the phantom was further enhanced by optimizing the adipose tissue layer to match the hardness values reported in human adipose tissue. Values from the literature range between 25.7 and 35.0 Shore OO^23^^,^^42^ and among the tested formulations, the silicone without deadener most closely matched this range, with a measured hardness of 31.2±1.9 Shore OO. Although the manufacturer’s technical bulletin for Platsil Gel 00 reports a cured hardness of Shore OO–30,^60^ it was important for the purposes of this study to experimentally verify this claim. Verification was performed using an AD-100-OO Precision Shore Durometer, following the testing procedure outlined in ASTM D2240-15.^43^ Finally, this formulation was selected to replicate the mechanical properties of adipose tissue in the finger.

Optical characterization was a major focus of the study. The absorption and reduced scattering coefficients of both the adipose tissue and the interchangeable skin layers were measured and compared to reported human data.^44^^–^^50^ Across the three PPG wavelengths: green (530 nm), red (655 nm), and IR (940 nm), the phantom showed optical properties within physiologically relevant ranges. The three skin tones also visually corresponded to realistic pigmentation along the Fitzpatrick scale. In addition, to further support the anatomical realism of the model, the skin layers were shaped as finger “gloves,” allowing them to be placed around the phantom, which presents advantages for future testing of sensors such as pulse oximeters.

It is worth noting that the adipose tissue exhibited lower absorption in the green wavelength region compared with the adipose tissue Supplementary Material references. This may have lowered the absorption and likely contributed to the reduced amplitude of the green PPG signal, as smaller changes in absorption during the blood pulse produce smaller variations in the detected light intensity. By contrast, the slightly higher scattering observed in the adipose tissue phantom would have redirected more light back toward the sensor, which may have partially affected the waveform shape and amplitude of the measured PPG.

The integration of the phantom into an in vitro cardiovascular loop system enabled simulation of a pulsatile flow (60 bpm, 8  L/min, ∼90  mmHg). Reflectance-mode PPG signals were acquired at all three wavelengths using a custom-made finger clip probe with a 4-mm source-detector separation. A 10-s window of the filtered signals, shown in Fig. 10, where a visual reduction in signal amplitude can be clearly observed across increasing skin pigmentation, following the expected trend. However, for the green wavelength (530 nm), the PPG signal was entirely lost in both the medium and dark skin layers. This is likely due to insufficient surface perfusion in the finger phantom combined with the strong optical absorption of the green light by these skin layers, which may have affected the photodetector from capturing any signal.^22^

The extraction of four signal features was performed: peak-to-peak amplitude, SNR, AUC, and AC/DC ratio across all skin tones. These features were summarized using median and IQR and visualized through box plots in Fig. 11. The statistical significance of differences between skin tones was analyzed using non-parametric methods. Kruskal-Wallis tests followed by Dunn-Sidak pairwise comparisons revealed significant differences between skin tones. Although signals were recorded across all three wavelengths, statistical analysis was performed only for the red (655 nm) and IR (940 nm) wavelengths. This is because at the green wavelength (530 nm), PPG waveforms were only observed in pale skin signals, whereas in medium and dark skin tones, the signal resembled noise and no pulse beats could be detected. The significant drop in SNR, peak-to-peak amplitude, AUC, and AC/DC ratio in these skin tones aligns with previous reports,^7^^,^^61^ which highlighted the dominant optical absorption of melanin in the visible spectrum, where higher melanin content led to greater attenuation of incident light, limiting penetration depth and distorting signal morphology. The green wavelength, which primarily interacts with superficial layers, was most affected by the skin layer absorption. Although red and IR light demonstrated preserved PPG signals across all skin tones, these showed a decreasing trend in amplitude, SNR, and AC/DC ratio in darker pigmentation, which was an expected result.

Although the developed phantom successfully reproduced pulsatile optical signals, it is important to note that the blood–mimicking fluid used in this study exhibits a monotonic absorption spectrum, unlike real blood, which has distinct haemoglobin absorption peaks at 420, 540, and 580 nm.^62^ As such, the phantom is best suited for assessing single-wavelength PPG performance, and this behavior should be taken into account when comparing the performance of PPG signals between wavelengths, as it does not fully replicate the wavelength-specific absorption characteristics of hemoglobin that occur *in vivo*.

The loss of the green light PPG signal observed in darker skin tones represents an important limitation. In physiology, green light is highly sensitive to blood volume changes because haemoglobin strongly absorbs in this region. However, in the phantom, the absence of these haemoglobin absorption peaks, combined with the increased melanin absorption in dark skin layers, led to a reduction in the detected green light PPG. Another limitation in the phantom design is the thickness of the skin layer, which may not fully replicate the anatomical characteristics of human epidermal skin. Because green light primarily interacts with superficial layers, a thinner skin would better reproduce the optical path and light interaction occurring *in vivo*. In addition, the lack of more superficial vasculature is another limitation that may contribute to the reduced green PPG signal. The current vessel was positioned ∼2.5  mm below the skin surface, which is considerably deeper than *in vivo* vascular plexus, thereby reducing the penetration and sensitivity of short wavelengths as green light. Incorporating capillary-level vessel structures near the surface could help mimic the highly perfused nature of the fingertip and improve the acquired PPG signal. In addition, the reduced green light absorption in the adipose layer may further explain the diminished signal, as higher absorption during the blood pulse would produce a larger change in received light intensity. Similarly, the elevated scattering in the adipose tissue influences how light is redirected during the pulse, affecting the overall signal detected by the sensor. Addressing these limitations is a key consideration for future work, allowing more comprehensive and realistic testing of PPG in a wider selection of skin tones.

This study contributes to a growing body of literature on phantom-based evaluation of optical biosensors.^25^^–^^29^ Although previous work has simulated skin pigmentation by adding absorbers to silicone, this study advances the field by demonstrating how increased skin absorption directly impacts specific PPG features in a controlled and dynamic vascular finger phantom setup. The reported differences in median and IQRs for features such as SNR, amplitude, AUC, and AC/DC ratio across wavelengths and skin tones offer a quantitative insight into the optical performance limitations under varying skin pigmentation conditions.

Overall, this study contributes valuable insights for the continued development of PPG phantoms, particularly those aimed at evaluating the effects of skin pigmentation. It serves as a foundation for testing optical sensors under controlled and inclusive conditions. However, the phantom still requires further refinements. Future work should focus on improving superficial perfusion to better replicate capillary blood flow, particularly for wavelengths like green light that interact primarily with shallow tissue layers. In addition, characterizing the optical properties of the blood-mimicking fluid will be essential to ensure it accurately represents physiological conditions. These enhancements will provide a more realistic assessment of the PPG technology performance across diverse skin tones.

## 5 Conclusion

This study presented the design, development, and evaluation of a pulsatile vascular finger phantom with anatomically, mechanically, and optically realistic properties, which aimed to investigate the influence of skin pigmentation on PPG signals. By incorporating optically characterized interchangeable skin layers representing different Fitzpatrick tones and simulating physiological pulsatile flow, the phantom demonstrated the ability to produce PPG signals. Results showed a significant reduction and complete loss of the green (530 nm) PPG signal in medium and dark skin tones, attributed to the skin’s high absorption and limited superficial perfusion. Red (655 nm) and IR (940 nm) wavelengths performed more consistently; however, signal quality still decreased with darker skin tones. Statistical analysis confirmed significant differences in key features such as amplitude, SNR, AUC, and AC/DC ratio across skin tones, particularly at the red wavelength. These findings highlight the need for inclusive design and validation of optical health technologies across all populations.

This vascular finger phantom provides a valuable tool for benchmarking optical sensors and signal processing algorithms under controlled and repeatable conditions that can mitigate the impact of skin pigmentation on PPG signal quality. Future improvements should focus on enhancing superficial perfusion by incorporating capillary-level structures and refining the optical properties of both the adipose tissue and blood-mimicking fluid to more closely match physiological values. These enhancements will enable more realistic simulation of PPG signals across all wavelengths and populations, supporting the development of more accurate and inclusive optical health monitoring technologies.

## Supplementary Material

10.1117/1.JBO.30.11.117002.s01

## Data Availability

Data produced in this study are available upon reasonable request.

## References

[1] Sjoding M. W. et al., “Racial bias in pulse oximetry measurement,” New Engl. J. Med. 383(25), 2477–2478 (2020). NEJMBH 10.1056/NEJMc2029240 33326721 PMC7808260

[r2] Wong A. K. I. et al., “Analysis of discrepancies between pulse oximetry and arterial oxygen saturation measurements by race and ethnicity and association with organ dysfunction and mortality,” JAMA Network Open 4(11), 1–14 (2021). 10.1001/jamanetworkopen.2021.31674 PMC9178439 34730820

[r3] UK Government Department of Health & Social Care, “Review launched into the health impact of potential bias in medical devices 2021,” [Online], https://www.gov.uk/government/news/review-launched-into-the-health-impact-of-potential-bias-in-medical-devices (Accessed 14 March 2025).

[r4] Cabanas A. M. et al., “Skin pigmentation influence on pulse oximetry accuracy: a systematic review and bibliometric analysis,” Sensors 22(9), 3402 (2022). SNSRES 0746-9462 10.3390/s22093402 35591092 PMC9102088

[5] Colvonen P. J., “Response to: investigating sources of inaccuracy in wearable optical heart rate sensors,” NPJ Digit. Med. 3, 18 (2021). 10.1038/s41746-020-0226-6 PMC7910598 33637822

[6] Kyriacou P. A. et al., “Inaccuracy of pulse oximetry with dark skin pigmentation: clinical implications and need for improvement,” Br. J. Anaesth. 130(1), E33–E36 (2023). BJANAD 0007-0912 10.1016/j.bja.2022.03.011 35430087

[7] Setchfield K. et al., “Effect of skin color on optical properties and the implications for medical optical technologies: a review,” J. Biomed. Opt. 29(01), 010901 (2024). JBOPFO 1083-3668 10.1117/1.JBO.29.1.010901 38269083 PMC10807857

[8] Okunlola O. E. et al., “Pulse oximeter performance, racial inequity, and the work ahead,” Respir. Care 67(2), 252–257 (2022). 10.4187/respcare.09795 34772785

[9] Bickler P. E. Feiner J. R. Severinghaus J. W., “Effects of skin pigmentation on pulse oximeter accuracy at low saturation,” Anesthesiology 102(4), 715–719 (2005). ANESAV 0003-3022 10.1097/00000542-200504000-00004 15791098

[r10] Vesoulis Z. et al., “Racial discrepancy in pulse oximeter accuracy in preterm infants,” J. Perinatol. 42(1), 79–85 (2022). JOPEEI 0743-8346 10.1038/s41372-021-01230-3 34642469 PMC8508473

[r11] Henry N. R. et al., “Disparities in hypoxemia detection by pulse oximetry across self-identified racial groups and associations with clinical outcomes,” Crit. Care Med. 50(2), 204–211 (2022). CCMDC7 0090-3493 10.1097/CCM.0000000000005394 35100193 PMC9070439

[r12] Philip K. E. J. Tidswell R. McFadyen C., “Racial bias in pulse oximetry: more statistical detail may help tackle the problem,” Br. Med. J. 372, n298 (2021). BMJOAE 0007-1447 10.1136/bmj.n298 33531354

[r13] Ebmeier S. J. et al., “A two centre observational study of simultaneous pulse oximetry and arterial oxygen saturation recordings in intensive care unit patients,” Anaesth. Intens. Care 46(3), 297–303 (2018). ANIMD2 0171-1814 10.1177/0310057X1804600307 29716488

[r14] Zeballos R. J. Weisman I. M., “Reliability of noninvasive oximetry in black subjects during exercise and hypoxia,” Am. Rev. Respir. Dis. 144(6), 1240–1244 (1991). ARDSBL 0003-0805 10.1164/ajrccm/144.6.1240 1741533

[r15] Jubran A. Tobin M. J., “Reliability of pulse oximetry in titrating supplemental oxygen therapy in ventilator-dependent patients,” Chest 97(6), 1420–1425 (1990). CHETBF 0012-3692 10.1378/chest.97.6.1420 2347228

[r16] Ries A. L. Prewitt L. M. Johnson J. J., “Skin color and ear oximetry,” Chest 96(2), 287–290 (1989). CHETBF 0012-3692 10.1378/chest.96.2.287 2752811

[r17] Fawzy A. et al., “Skin pigmentation and pulse oximeter accuracy in the intensive care unit: a pilot prospective study,” Am. J. Respir. Crit. Care Med. 210(3), 355–358 (2024). 38820169 10.1164/rccm.202401-0036LE PMC11348963

[r18] Crooks C. J. et al., “Differential pulse oximetry readings between ethnic groups and delayed transfer to intensive care units,” QJM: Int. J. Med. 116(1), 63–67 (2023). 10.1093/qjmed/hcac218 PMC9928225 36066450

[r19] Mettler S. et al., “Clinical factors associated with racial differences in the prevalence of occult hypoxemia: a retrospective case-control study,” Eur. Respiratory J., 64(68), PA2614 (2024). 10.1183/13993003.congress-2024.PA2614

[r20] Patwari N. Huang D. Bonetta-Misteli F., “Racial disparities in pulse oximetry cannot be fixed with race-based correction,” in Proc. - 2023 IEEE/ACM Int. Conf. Connected Health: Appl., Syst. and Eng. Technol., CHASE 2023, pp. 143–147 (2023). 10.1145/3580252.3586986

[21] Valbuena V. S. M. Merchant R. M. Hough C. L., “Racial and ethnic bias in pulse oximetry and clinical outcomes,” JAMA Intern. Med. 182(7), 699–700 (2022). 10.1001/jamainternmed.2022.1903 35639397

[22] Kyriacou P. Chatterjee S., “The origin of photoplethysmography,” in Photoplethysmography: Technology, Signal Analysis and Applications, Kyriacou P. Allen J., Eds., Elsevier, pp. 17–42 (2021).

[23] Nomoni M. May J. M. Kyriacou P. A., “Novel polydimethylsiloxane (PDMS) pulsatile vascular tissue phantoms for the in-vitro investigation of light tissue interaction in photoplethysmography,” Sensors 20(15), 4246 (2020). SNSRES 0746-9462 10.3390/s20154246 32751541 PMC7435705

[24] Pogue B. W. Patterson M. S., “Review of tissue simulating phantoms for optical spectroscopy, imaging and dosimetry,” J. Biomed. Opt. 11(4), 041102 (2006). JBOPFO 1083-3668 10.1117/1.2335429 16965130

[25] Afshari A. et al., “Evaluation of the robustness of cerebral oximetry to variations in skin pigmentation using a tissue-simulating phantom,” Biomed. Opt. Express 13(5), 2909 (2022). BOEICL 2156-7085 10.1364/BOE.454020 35774336 PMC9203096

[r26] Bhusal A. et al., “Development and characterization of silicone-based tissue phantoms for pulse oximeter performance testing,” J. Biomed. Opt. 29(S3), S33314 (2025). JBOPFO 1083-3668 10.1117/1.JBO.29.S3.S33314 PMC11706025 39776836

[r27] Jenne S. Zappe H., “Multiwavelength tissue-mimicking phantoms with tunable vessel pulsation,” J. Biomed. Opt. 28(04), 045003 (2023). JBOPFO 1083-3668 10.1117/1.JBO.28.4.045003 37077500 PMC10109273

[r28] Vogt W. C. Wear K. A. Pfefer T. J., “Phantoms for evaluating the impact of skin pigmentation on photoacoustic imaging and oximetry performance,” Biomed. Opt. Express 14(11), 5735 (2023). BOEICL 2156-7085 10.1364/BOE.501950 38021140 PMC10659791

[29] Swamy S. K. N. et al., “Pulse oximeter bench tests under different simulated skin tones,” Med. Biol. Eng. Comput. 63, 1931–1942 (2024). MBECDY 0140-0118 10.1007/s11517-024-03091-2 38653879 PMC12204950

[30] Tilley A. R., The Measure of Man and Woman: Human Factors in Design, Illustrated, Revised, Wiley, New York (2002).

[31] Strauch B. de Moura W., “Arterial system of the fingers,” J. Hand Surg. 15(1), 148–154 (1990). 10.1016/S0363-5023(09)91123-6 2299156

[32] Wilting J., “Integrated vascular anatomy,” in Pan Vascular Medicine: Integrated Clinical Management, Lanzer P. Topol E. J., Eds., pp. 50–75, Springer Berlin Heidelberg, Berlin, Heidelberg (2002).

[33] Boonya-ananta T. et al., “Synthetic photoplethysmography (PPG) of the radial artery through parallelized Monte Carlo and its correlation to body mass index (BMI),” Sci. Rep. 11(1), 1–11 (2021). SRCEC3 2045-2322 10.1038/s41598-021-82124-4 33510428 PMC7843978

[34] Camasão D. B. Mantovani D., “The mechanical characterization of blood vessels and their substitutes in the continuous quest for physiological-relevant performances. A critical review,” Mater. Today Bio 10, 100106 (2021). 10.1016/j.mtbio.2021.100106 PMC8050780 33889837

[35] Avolio A. P., “Multi-branched model of the human arterial system,” Med. Biol. Eng. Comput. 18(6), 709–718 (1980). MBECDY 0140-0118 10.1007/BF02441895 7230917

[36] Khamdaeng T. et al., “Arterial stiffness identification of the human carotid artery using the stress-strain relationship in vivo,” Ultrasonics 52(3), 402–411 (2012). ULTRA3 0041-624X 10.1016/j.ultras.2011.09.006 22030473 PMC4009743

[37] Langewouters G. J. et al., “Pressure-diameter relationships of segments of human finger arteries,” Clin. Phys. Physiol. Meas. 7, 43 (1986). CPPMD5 0143-0815 10.1088/0143-0815/7/1/003 3956118

[38] Nomoni M. May J. M. Kyriacou P. A., “Fabricating novel PDMS vessels for phantoms in photoplethysmography investigations,” in Proc. Annu. Int. Conf. IEEE Eng. in Med. and Biol. Soc., EMBS, pp. 4458–4461 (2020). 10.1109/EMBC44109.2020.9176476 33018984

[39] Karimpour P. et al., “Customisable silicone vessels and tissue phantoms for in vitro photoplethysmography investigations into cardiovascular disease,” Sensors 24(5), 1681 (2024). SNSRES 0746-9462 10.3390/s24051681 38475217 PMC10933982

[40] Khder Y. et al., “Effects of blood pressure control on radial artery diameter and compliance in hypertensive patients,” Am. J. Hypertens. 10(3), 269–274 (1997). AJHYE6 0895-7061 10.1016/S0895-7061(96)00347-0 9056683

[41] Nwafor C. I. et al., “Assessment of a noninvasive optical photoplethysmography imaging device with dynamic tissue phantom models,” J. Biomed. Opt. 22(09), 096003 (2017). JBOPFO 1083-3668 10.1117/1.JBO.22.9.096003 28895317

[42] Falanga V. Bucalo B., “Use of a durometer to assess skin hardness,” J. Am. Acad. Dermatol. 29(1), 47–51 (1993). JAADDB 0190-9622 10.1016/0190-9622(93)70150-R 8315077

[43] ASTM International, “ASTM2240-15: Standard test method for rubber property—durometer hardness,” ASTM International, West Conshohocken, Pennsylvania (2021).

[44] Shimojo Y. et al., “Measurement of absorption and reduced scattering coefficients in Asian human epidermis, dermis, and subcutaneous fat tissues in the 400- to 1100-nm wavelength range for optical penetration depth and energy deposition analysis,” J. Biomed. Opt. 25(04), 045002 (2020). JBOPFO 1083-3668 10.1117/1.JBO.25.4.045002 32356424 PMC7191311

[r45] Salomatina E. et al., “Optical properties of normal and cancerous human skin in the visible and near-infrared spectral range,” J. Biomed. Opt. 11(6), 064026 (2006). JBOPFO 1083-3668 10.1117/1.2398928 17212549

[r46] Bashkatov A. N. et al., “Optical properties of human skin, subcutaneous and mucous tissues in the wavelength range from 400 to 2000 nm,” J. Phys. D Appl. Phys. 38(15), 2543–2555 (2005). 10.1088/0022-3727/38/15/004

[r47] Simpson C. R. et al., “Near-infrared optical properties of ex vivo human skin and subcutaneous tissues measured using the Monte Carlo inversion technique,” Phys. Med. Biol. 43(9), 2465–2478 (1998). PHMBA7 0031-9155 10.1088/0031-9155/43/9/003 9755939

[r48] Peters V. G. et al., “Optical properties of normal and diseased human breast tissues in the visible and near infrared,” Phys. Med. Biol. 35(9), 1317–1334 (1990). PHMBA7 0031-9155 10.1088/0031-9155/35/9/010 2236211

[r49] Tseng S. Grant A. Durkin A. J., “In vivo determination of skin-infrared optical properties using diffuse optical spectroscopy,” J. Biomed. Opt. 13(1), 014016 (2008). JBOPFO 1083-3668 10.1117/1.2829772 18315374 PMC2626348

[50] Saager R. B. et al., “In vivo measurements of cutaneous melanin across spatial scales: using multiphoton microscopy and spatial frequency domain spectroscopy,” J. Biomed. Opt. 20(6), 066005 (2015). JBOPFO 1083-3668 10.1117/1.JBO.20.6.066005 26065839 PMC4463032

[51] Sarid H. Abookasis D., “Extraction of the anisotropy factor and refractive index of biological tissue in the near-infrared region from diffusion approximation in the spatial frequency domain,” Opt. Commun. 508, 127749 (2022). OPCOB8 0030-4018 10.1016/j.optcom.2021.127749

[52] Bashkatov A. N. Genina E. A. Tuchin V. V., “Optical properties of skin, subcutaneous, and muscle tissues: a review,” J. Innov. Opt. Health Sci. 4(1), 9–38 (2011). 10.1142/S1793545811001319

[53] Lualdi M. et al., “A phantom with tissue-like optical properties in the visible and near infrared for use in photomedicine,” Lasers Surg. Med. 28(3), 237–243 (2001). LSMEDI 0196-8092 10.1002/lsm.1044 11295758

[54] Erbel R. Eggebrecht H., “Aortic dimensions and the risk of dissection,” Heart 92(1), 137–142 (2006). 10.1136/hrt.2004.055111 16365370 PMC1861012

[55] Dotter C. T. Roberts D. J. Steinberg I., “Aortic length: angiocardiographic measurements,” Circulation 2(6), 915–920 (1950). CIRCAZ 0009-7322 10.1161/01.CIR.2.6.915 14783846

[56] Al Talalwah W. Getachew D. Soames R., “Morphological feature of brachial artery and its clinical significance,” J. Morphol. Sci. 32(3), 129–134 (2015). 10.4322/jms.079014

[57] Bidarkotimath S. Avadhani R. Kumar A., “Primary pattern of arteries of upper limb with relevance to their variations,” Int. J. Morphol. 29(4), 1422–1428 (2011). IJIMBQ 0020-7322 10.4067/S0717-95022011000400059

[58] Beniwal S. Bhargava K. Kausik S. K., “Size of distal radial and distal ulnar arteries in adults of southern Rajasthan and their implications for percutaneous coronary interventions,” Indian Heart J. 66(5), 506–509 (2014). 10.1016/j.ihj.2014.08.010 25443603 PMC4223202

[59] May J., “Investigation of fontanelle photoplethysmographs and oxygen saturations in intensive care neonates and infants utilising miniature photometric sensors,” Doctoral thesis, Unpublished, City University of London (2013).

[60] Polytek Development Corp., “PlatSil silicone gels: technical bulletin,” 2024, Easton, Pennsylvania, https://polytek.com/content/pdf/TDS/PlatSilGels_TechnicalBulletin_Polytek_ALL_SkinSafe.pdf (accessed 19 November 2025).

[61] Setchfield K. et al., “Relevance and utility of the in-vivo and ex-vivo optical properties of the skin reported in the literature: a review [Invited],” Biomed. Opt. Express 14(7), 3555 (2023). BOEICL 2156-7085 10.1364/BOE.493588 37497524 PMC10368038

[62] Prahl S., “Optical absorption of hemoglobin,” Oregon Medical Laser Center, 1999, https://omlc.org/spectra/hemoglobin/ (accessed 19 November 2025).

