# Supplementary material for: Evaluation of skin pigmentation effect on photoplethysmography signals using a vascular finger phantom with tunable optical and mechanical properties
Source: J Biomed Opt. 2025 Nov 25;30(11):117002. doi: 10.1117/1.JBO.30.11.117002 (PMC12646468; doi:10.1117/1.JBO.30.11.117002)
Supplement: Supplementary file 1 [file JBO_030_117002_SD001.pdf]

A literature review was conducted to determine the absorption (μ_a) and reduced scattering coefficients (μ'_s) of adipose tissue. Table S1 summarizes the findings, outlining key studies that have measured the optical properties of adipose tissue using different experimental approaches. It provides details on measurement sites and techniques, instrumentation, sample characteristics, wavelength range, study objectives, blood content status, sample preparation methods, and anisotropy factor used. The table highlights the methodological differences that may influence the comparability of optical data across the studies. It is important to note that, while other papers may report optical properties of adipose tissue, only those studies that included a wavelength range covering 500 nm to 1000 nm were included, as this is the range of primary interest for the current review.

Table S1. Summary of reported studies on the optical properties of human adipose tissue: methods, sample preparation, and key parameters

Author, Year	Measurement site (measurement technique)	Measurement Instrument	N samples (skin colour/ ethnic group)	Wavelength range [nm]	Aim	Blood	Sample storage and preparation methods	Anisotropy factor
**Shimojo et al, 2020** [44]	Face, abdomen, thigh, axilla, clavicle, and ear (double integrating sphere diffuse reflectance spectroscopy)	Double integrating spheres (CSTM-3P-GPS-033SL, Labsphere), spectrometer (MAYP10161, Maya2000-Pro, Ocean Optics), optical fibre (CUSTOM-PATCH-2243142, Ocean Optics), xenon lamp (L2273 and C8849, Hamamatsu Photonics, Japan), reflectance standard (SRS-20-010, SRS-99-010, Labsphere)	15 samples subcutaneous fat (Asian)	400 - 1100	Measure absorption and scattering coefficients of Asian epidermis, dermis, and subcutaneous fat for evaluating optical penetration depth and energy deposition	No	Immersed in saline solution, stored at 4°C, measured after maximum 12 hours defreezing	0.9
**Salomatina et al, 2006** [45]	Face, scalp, neck, and back (integrating sphere spectrophotometer)	Single integrating sphere (4P-GPS-033-SL, Lab- sphere), spectrometer (HR2000, Ocean Optics) (EPP2000-NIR, StellarNet), hallogen lamp (HL- 2000-HP-FHSA, Ocean Optics)	10 samples subcutaneous fat (Caucasian)	370 - 1600	Determine and compare absorption and scattering properties of cancerous and healthy skin layers	Yes	Immersed in saline solution, measured after maximum 7 hours of excision	0.8
**Bashkatov et al, 2005** [46]	Peritoneum area (integrating sphere spectrophotometer)	Spectrophotometer (CARY-2415, Varian, Australia), integrating sphere, halogen lamp	6 samples	400 - 2000	Measure absorption and scattering coefficients of human skin, subcutaneous adipose tissue and mucous in the wavelength range from 400 to 2000 nm	Yes	Immersed in saline solution at 20°C, measured 3-4 hours after biopsy	0.9
**Simpson et al, 1998** [47]	Abdominal and breast tissue (integrating sphere spectrophotometer)	Integrating sphere (LabSphere RT-060-SF), optical fibre-coupled white light, spectrophotometer, 50% reflectance standard (LabSphere SRS-50-010) w	12 samples (Caucasian)	620 - 1000	Measure the absorption and scattering coefficients of human dermis, subdermis, and muscle in the wavelength range from 620 to 1000 nm.	No	Refrigerated for 5 days and then returned to room temperature	0.9
**Peters et al, 1990** [48]	Breast tissue (integrating sphere)	Integrating sphere, xenon arc lamp, monochromator, reflectance standard (Labsphere Inc., North Sutton, New Hampshire)	7 samples	500 - 1100	Determine optical absorption and scattering coefficients of diseased and normal breast tissue	Yes	Frozen and then measured	0.945 – 0.985

A literature review was conducted to determine the absorption (μ_a) and reduced scattering coefficients (μ'_s) of skin. The selection criteria focused on studies that classified optical properties into at least two groups, using the Fitzpatrick scale or ethnicity, as these are the most common skin classification methods. Table S2 summarizes the findings, outlining key studies that have measured the optical properties of skin in different skin colour groups using different experimental approaches. It provides details on study type, measurement sites and techniques, instrumentation, sample characteristics, wavelength range and study objectives. Notably, Table S2 highlights measurement sites, as optical property variations can arise due to differences in pigmentation across body regions, influenced by factors such as sun exposure, and epidermal thickness [49].

Table S2. Summary of reported studies on the optical properties of different human skin colour groups: methods, sample groups and key parameters

Author, Year	Type study	Measurement site (measurement technique)	Measurement Instrument	N participants (skin colour classification)	Wavelength range [nm]	Aim
**Saager et al, 2015** [50]	*in vivo*	Dorsal forearm and upper inner arm (spatial-frequency domain spectroscopy)	Quartz-Tungsten-Halogen light source (Moritex, MHF-D100LR), Spectrometer (Oriel 77480),	12 healthy subjects (Fitzpatrick Skin types II, III, IV, VI)	450 – 1000	Quantify in vivo melanin volume fraction and epidermal and melanin distribution thickness
**Tseng et al, 2008** [49]	*in vivo*	Volar forearm and palm (fibre optic diffuse reflectance spectroscopy)	Tungsten halogen light source (Ocean Optics, Model HL 2000), spectrometer (BWTek, Model 611), bank of frequency modulated diodes	15 subjects (5 African descendent, 5 Asians, 5 Caucasians)	650 – 1000	Develop a diffuse optical spectroscopic probe to determine the optical properties of in vivo human skin
**Simpson et al, 1998** [47]	*ex vivo*	Abdominal and breast tissue (single integrating sphere diffuse reflectance spectroscopy)	Double integrating spheres (CSTM-3P-GPS- 033SL, Labsphere), spectrometer (MAYP10161, Maya2000-Pro, Ocean Optics), optical fibre (CUSTOM-PATCH-2243142, Ocean Optics), xenon lamp (L2273 and C8849, Hamamatsu Photonics, Japan), reflectance standard (SRS-20-010, SRS-99-010, Labsphere)	Samples from 5 subjects (4 Caucasians, 1 Black)	620 - 1000	Measure the absorption and scattering coefficients of human dermis, subdermis, and muscle using a Monte Carlo inversion technique
